# Inhibitory Effects of Sodium Arsenite and Acacia Honey on Acetylcholinesterase in Rats

**DOI:** 10.1155/2015/903603

**Published:** 2015-03-02

**Authors:** Aliyu Muhammad, Oyeronke A. Odunola, Michael A. Gbadegesin, Abdullahi B. Sallau, Uche S. Ndidi, Mohammed A. Ibrahim

**Affiliations:** ^1^Department of Biochemistry, Cancer Research and Molecular Biology Laboratories, University of Ibadan, Ibadan, Oyo State, Nigeria; ^2^Department of Biochemistry, Ahmadu Bello University, Zaria, Kaduna State, Nigeria

## Abstract

This study was conducted to investigate the effect of sodium arsenite and Acacia honey on acetylcholinesterase (AChE) activity and electrolytes in the brain and serum of Wistar rats. Male Wistar albino rats in four groups of five rats each were treated with distilled water, sodium arsenite (5 mg/kg body weight), Acacia honey (20% v/v), and sodium arsenite and Acacia honey, daily for one week. The sodium arsenite and Acacia honey significantly (*P* < 0.05) decreased AChE activity in the brain with the combined treatment being more potent. Furthermore, sodium arsenite and Acacia honey significantly (*P* < 0.05) decreased AChE activity in the serum. Strong correlation was observed between the sodium and calcium ion levels with acetylcholinesterase activity in the brain and serum. The gas chromatography mass spectrometry analysis of Acacia honey revealed the presence of a number of bioactive compounds such as phenolics, sugar derivatives, and fatty acids. These findings suggest that sodium arsenite and/or Acacia honey modulates acetylcholinesterase activities which may be explored in the management of Alzheimer's diseases but this might be counteracted by the hepatotoxicity induced by arsenics.

## 1. Introduction

Arsenic compounds are ubiquitous in nature and are released into the environment via industrial or agricultural processes as well as some medical applications [1]. Consumption of arsenicals such as sodium arsenite through contaminated water is prevalent in many areas of the world [2]. Sodium arsenite is a clastogen causing chromosomal breakage [3], which interacts with other substances like metals to potentiate its effects [4]. Its administration has been reported to compromise the integrity of the liver of mouse, rat, fish, and goat [5–7]. However, administration of sodium arsenite induces oxidative stress with severe demyelination and other morphological alterations in axons of peripheral nerves which may potentially induce changes in the generation and distribution of action potentials in peripheral nerves, thereby causing an impediment in transmission of nerve impulses [8]. It has been reported that arsenic can act as comutagen because of its ability to bind and inhibit the activities of thiol containing enzymes [9], such as DNA ligase [10] causing defects during DNA replication/repair, recombination, and joining of single- and double-stranded DNA breaks [11].

Alzheimer's disease is a progressive dementing neurodegenerative disorder in elderly, which is pathologically characterized by the presence of senile plaques and neurofibrillary changes in the brains of affected individuals [12]. High activities of acetylcholinesterase (EC 3.1.1.7) (AChE) in the brain have been implicated in the pathogenesis of the disease and its inhibition is considered as a viable therapeutic strategy in the management of the disease [13]. The main biochemical role of AChE is the termination of impulse transmission at cholinergic synapses by rapid hydrolysis of the neurotransmitter, acetylcholine (ACh) [13]. AChE exhibits a high specific activity similar to that of a diffusion-controlled reaction [14].

Millions of people around the world get exposed to high levels of arsenic compounds in drinking-water which often largely affect rural dwellers. Considering the adverse effects of these arsenic compounds on the nervous system and the high prevalence of Alzheimer's disease among such rural dwellers, it is worthwhile to investigate the effects of the arsenic compounds on the pathophysiology of Alzheimer's disease. Unfortunately, however, such an important relationship has not been previously investigated either in humans or experimental animals.

On the other hand, most of the arsenics-exposed individuals as well as the Alzheimer's patients are frequently exposed to a number of functional foods. One of such functional foods is honey which has prophylactic and curative properties. Honey is basically a supersaturated solution of sugars, produced by honeybees via a regurgitation mechanism of plant parts [15–18] with fructose and glucose as the most abundant sugars present in it. Variety of constituents such as phenolic acids, flavonoids, enzymes, carotenoids, organic acids, and proteins have been reported to be present in honey [19–21]. In addition to the above-mentioned constituents, other bioactive compounds such as vitamins, antioxidants, and hydrogen peroxide are also reported to be present in honey [22]. Thus, honey as a natural source of antioxidant might reduce the risk of Alzheimer's disease because of the crucial role of oxidative stress in the pathogenesis of the disease [23]. It has also been documented that honey exhibits several medicinal properties which include antitumor, antimetastatic, and antiangiogenic effects [24]. Others are antibacterial, anti-inflammatory, immune-stimulant, antiulcer, and wound/burn healing properties [25]. Acacia honey is a type of honey produced by bees from the Acacia flowers, hence, the name. Earlier report from our laboratory demonstrated that daily administrations of Acacia honey to Wistar rats have some biological effects on the clinical and biochemical parameters [18]. We have also demonstrated its antiproliferative effects against prostate cancer cell line [16] and lung cancer cell line* in vitro* [17]. Furthermore, we have evidently demonstrated the fact that fractionation of Acacia honey negatively affected its antioxidant potentials by making it a radical generating agent in contrast to the unfractionated sample. In fact, the antioxidant potential of the whole Acacia honey was comparable to *α*-tocopherol; a well-known standard antioxidant [15].

However, the effects of honey on Alzheimer's disease have not been fully investigated especially with respect to the possible modulation of heavy-metals (arsenics) associated toxicity during the disease. Hence, this study was conducted to investigate the possible effects of arsenic compounds (sodium arsenite) on the pathology of Alzheimer's disease as well as the modulatory role of Acacia honey on the heavy-metals (arsenics) associated toxicity during the disease.

## 2. Results

**Figure 1:**
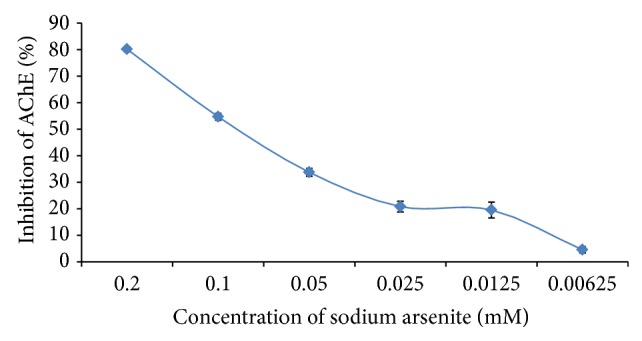
The effect of sodium arsenite on the activity of acetylcholinesterase* in vitro*. Values are presented as mean ± SD.

**Figure 2:**
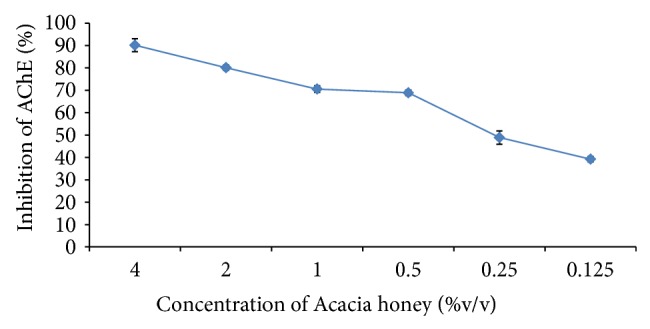
The effect of Acacia honey on the activity of acetylcholinesterase* in vitro*. Values are presented as mean ± SD.

In the* in vitro* study, Acacia honey and sodium arsenite demonstrated potent inhibition of AChE in a concentration-dependent pattern (Figures 1 and 2) with an exceptional IC_50s_ of 0.26% (v/v) and 0.0885 mM, respectively. Interestingly, sodium arsenite also significantly (*P* < 0.05) inhibited AChE in the brain of the experimental animals but Acacia honey had a more significant inhibition of the brain AChE than sodium arsenite (Table 1). However, among all the treatments, the combined administration of Acacia honey and sodium arsenite demonstrated the most potent inhibition of the brain AChE (Table 1). There was no significant difference (*P* < 0.05) in the serum levels of AChE among the treatment groups (Table 1).

A significant (*P* < 0.05) decrease in the brain Ca^2+^ and Na^+^ levels was observed in the sodium arsenite and Acacia honey treated groups and the reduction in the brain levels of these electrolytes was more significant (*P* < 0.05) in the coadministered group (Table 2). There was no significant (*P* > 0.05) difference across all the groups in the brain K^+^ levels. All the treatments significantly (*P* < 0.05) decreased the brain levels of Cl^−^ but there was no significant (*P* > 0.05) difference among the treated groups compared to the control (Table 2). There was no significant (*P* > 0.05) difference across all the groups in the electrolyte levels in serum except when compared with control (*P* < 0.05) (Table 3).

Bearing in mind the role of ions in the release of acetylcholine and* vice versa*, the correlation between AChE activity and electrolyte levels in brain and serum was calculated with a strong correlation between Ca^2+^ and Na^+^ levels and AChE activity in the brain (*R* ^2^ = 0.962 and 0.838) with no correlation in terms of K^+^ and Cl^−^ levels. However, no strong correlations were observed between AChE activity and electrolyte level in serum.

**Figure 3:**
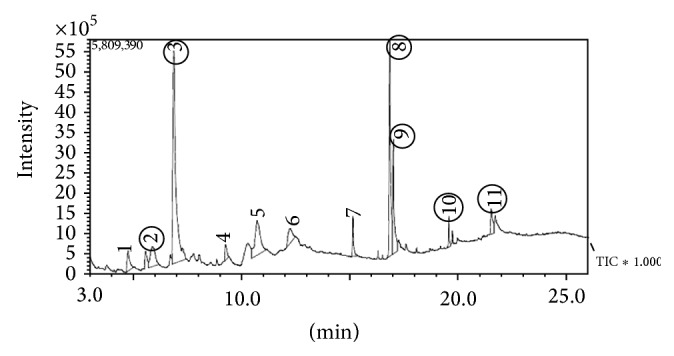
GC-MS analysis results of Acacia honey.

**Figure 4:**
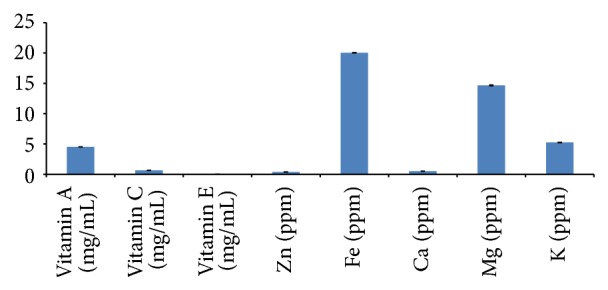
The vitamin and mineral contents of Acacia honey. The mineral element contents of Acacia honey. Values are presented as mean ± SD.

The GC-MS chromatogram of the Acacia honey is presented in Figure 3 and the list of proposed bioactive components was presented in Table 4.  Figure 4 shows the vitamin contents of Acacia honey with vitamin A being highest in content followed by vitamin C. Some important mineral elements (Figure 4) of physiological importance like calcium, iron, magnesium, potassium and zinc. The concentration of these elements in Acacia honey is in the order: iron > magnesium > potassium > calcium > zinc.

## 3. Discussion

Acetylcholine (ACh) is a neurotransmitter that functions in conveying nerve impulses across synaptic clefts within the central nervous system (CNS) [26]. Following the transmission of an impulse across the synapse by the release of ACh, AChE is released into the synaptic cleft [27]. This enzyme hydrolyzes ACh to choline and acetate, and transmission of the nerve impulse is terminated [28]. In this study, we report the effects of sodium arsenite and Acacia honey on acetylcholinesterase activity as well as the relationship with electrolyte levels.

Arsenic compounds are known to exert toxicity by binding and inactivating thiol groups in proteins [9] and this phenomenon could account for the observed* in vitro* inhibition of the AChE by the sodium arsenite. Interestingly, the sodium arsenite was also able to inhibit the AChE in the brain which might suggest that this compound possesses the ability to cross the blood-brain barrier and modulate the function of the enzyme via binding to the thiol groups of the protein. Also, the observed inhibitory effects of sodium arsenite on the brain AChE were potentiated by Acacia honey, which could be linked to the active ingredients in honey that are perhaps lipophilic in nature and, therefore, could increase the effective concentration of the sodium arsenite entering the brain. It is also possible that the potentiation of sodium arsenite inhibitory activity was due to the identified phenolic compounds such as p-hydroxybenzoic acid, cinnamic acid, and chrysin present in the Acacia honey which could also inhibit the AChE. Indeed, phenolics such as anacardic acids, cardols, cardanols, and methylcardols have been reported to inhibit AChE [29]. Overall, the foregoing observations suggest that sodium arsenite and/or Acacia honey could be beneficial in the management of Alzheimer's disease in this regard. Inhibition of AChE implies more of ACh in circulation, which has been reported to bind to angiotensin II, thereby preventing angiotensin II induced oxidative stress [30].

Serum cholinesterases presumably originate in liver cells but other organs also contribute to the pool of these enzymes in the plasma [31]. Activity of serum AChE could be an index of liver function and low activity of the enzyme has been reported in so many liver dysfunctions like jaundice and cirrhosis [3, 32]. Our findings indicated that the sodium arsenite and/or Acacia honey could lower the serum levels of AChE, though insignificantly, which seemingly indicate hepatotoxicity. Some identified components of Acacia honey such as pyrazol-3-one and 9-octadecanoic acid-2-hydroxy-1-(hydroxymethyl) ethyl ester as well as the sodium arsenite have been reported to be hepatotoxic [33, 34]. However, it is plausible to suggest that sodium arsenite and/or Acacia honey have some beneficial effects on hepatic cells but the inhibitory effects of the sodium arsenite as well as the identified components of the honey on thiol containing proteins led to a decline in the activity of the serum enzyme. Overall, information derived from the serum tends to suggest that these agents have some level of hepatotoxicity.

Balance in the levels of Ca^2+^ and Na^+^ in the brain and blood is a prerequisite for neurotransmission. Our results showed that the activity of AChE positively correlates with the levels of these ions. The role of Ca^2+^ and Na^+^ in neurotransmission in regulating the release of neurotransmitters and in the pathogenesis of neurological diseases has been investigated [35]. When presynaptic cell releases a brief pulse of ACh, both sites on the postsynaptic cell receptor are occupied briefly and the channel opens, which allow the passage of either Ca^2+^ or Na^+^. The inward flux of these ions depolarizes the plasma membrane, initiating subsequent events that vary with the type of tissue [36]. High concentration of Ca^2+^ and low concentration of Na^+^ are required for AChE release but the consistent decrease in AChE activity in this study means that there is more ACh than AChE present to facilitate the synaptic nerve transfer. Interestingly, sodium channels play a central role in action potential generation and are uniquely poised to influence the efficacy of transmitter release [37].

Oxidative processes have been implicated in the onset and development of degenerative diseases and foods rich in polyphenols [38], vitamins, and minerals [39] may have a nutritional and health beneficial effects based on our findings.

## 4. Conclusion

Data from this study suggest that exposure to sodium arsenite could be beneficial in the management of Alzheimer's disease; however, it seems toxic to hepatic tissues. Furthermore, Acacia honey could potentiate the action of sodium arsenite in both cases and therefore may be explored in the management of Alzheimer's disease.

## 5. Materials and Methods

### 5.1. Chemicals and Reagents

Sodium arsenite (5 mg/kg body weight) equivalent to two-tenth of the oral LD_50_ [40] was used in all experiments. Acacia honey was dissolved in distilled water to prepare a 20% (v/v) honey solution and 5 mL/kg body weight (b.w.) was used [18]. All other reagents and chemicals used were of analytical grade.

### 5.2. Sample Collection

Honey produced by* Apis mellifera* was collected from the North-West frontier of Pakistan during spring season of 2012 from* Acacia modesta* flower, identified, and maintained at 4°C until analysis, at the Industrial Analytical Centre, International Center for Chemical and Biological Sciences, University of Karachi, Karachi, Pakistan.

### 5.3. Determination of* In Vitro* Acetylcholinesterase Inhibitory Activity of Sodium Arsenite and Acacia Honey

The activity of AChE (Sigma-Aldrich) was determined spectrophotometrically by the modified method of Ellman's et al. [41] using acetylthiocholine iodide as substrate and 5-5′- dithiobis (2-nitrobenzoic) acid [DTNB] as a chromogen. Varying concentrations of sodium arsenite (0.2, 0.1, 0.05, 0.025, 0.0125, and 0.00625 mM) and honey (0.125, 0.25, 0.5, 1.0, 2.0, and 4.0% v/v) were used for inhibition studies, respectively. The reaction of DTNB with thiocholine released by the enzymatic hydrolysis of acetylthiocholine iodide was monitored at a wavelength of 412 nm. The percentage inhibition was calculated against the untreated enzymes and the IC_50_ determined.

### 5.4. Experimental Animals and Design

Twenty-four (24) male Wistar rats (150–195 g) were used in the present study. They were allowed to adapt for one week under standard laboratory conditions of 12 hr light-dark cycle before commencement of all experiments. The rats were maintained in the animal house of International Centre of Chemical and Biological Sciences, University of Karachi, Pakistan, with protocol for the study approved by the Institutional Animal Right Review Committee. During acclimatization, the rats were allowed free access to NIH-07 pelletized diet and water. In addition, all rats were catered in accordance with the National Institute of Health (NIH) Guide for the care and Use of Laboratory Animals. They were randomly grouped into four (4) groups of six (6) rats each. The animals were treated daily for seven days as shown in the experimental design below:

 Group 1: distilled water only;
 Group 2: 5 mg/kg b.w. sodium arsenite equivalent to 20% of oral LD_50_ [40];
 Group 3: 20% v/v Acacia honey + 5 mg/kg b.w. sodium arsenite;
 Group 4: 20% v/v Acacia honey at 5 mL/kg b.w.

### 5.5. Collection of Tissues and Blood Samples

Twenty-four (24) hrs after the last treatment, the rats were humanely sacrificed with sodium pentothal (60 mg/kg b.w.) after an overnight fast. The clotted blood samples were centrifuged at 3500 ×g at −4°C for 10 minutes to obtain the serum which was kept at −80°C until further analysis. The brain (cerebrum and cerebellum) and blood serum were also collected and the brain was homogenized in 1 : 5 of phosphate buffer (pH 7.4), centrifuged at 3500 ×g at 4°C for 10 minutes, and kept at −80°C until further analysis. Each time the supernatant/serum was outside the freezer, it was kept in ice bags. Using Auto Analyzer Hitachi Roche 7020 (902, Japan Inc.) the total protein contents of the serum and tissues were determined using the standard manufacturer's protocol.

### 5.6. Determination of Acetylcholinesterase Activity* In Vivo*

The activities of AChE in the blood serum and brain were determined spectrophotometrically by the method of Ellman's et al. [41] as modified by Srikumar et al. [42] using acetylthiocholine iodide as appropriate substrate and 5-5′- dithiobis (2-nitrobenzoic) acid [DTNB] as a chromogen. The reaction of DTNB with thiocholine released by the enzymatic hydrolysis of acetylthiocholine iodide was monitored at 412 nm. The specific activity of AChE was expressed as *μ*mole/gram of tissue/minute/mg protein for brain and *μ*mole/minute/mg protein for serum.

### 5.7. Determination of Electrolytes

Electrolytes levels were quantified in serum and brain tissues by using Auto Analyzer Hitachi Roche 7020 (902) according to manufacturer's protocols for calcium ion, whereas the levels of potassium, sodium, and chloride ions were determined by electrolyte analyzer (Ion Selective Electrode, China) according to manufacturer's protocol.

### 5.8. Qualitative Identification of the Proposed Active Principles of Acacia Honey

Gas chromatography/mass spectrometry (GC-MS) was carried out by Shimadzu GCMS-QP2010 PLUS Japan according to manufacturer's protocol.

### 5.9. Determination of Mineral Elements in Acacia Honey

The mineral elements: calcium, iron, potassium, magnesium, and zinc, were determined using Atomic Absorption Spectrometry (AAS) machine according to manufacturer's protocol.

### 5.10. Determination of Vitamins in Acacia Honey

Vitamins A, C, and E were determined in the honey after adopting the methods of Rutkowski and Grzegorczyk [43] and Dahot et al. [44].

### 5.11. Statistical Analysis

To address the biological variability and stability of the samples, each and every experiment was repeated at least three times and the results were expressed as mean ± standard deviation. Differences between the groups were analyzed by one-way analysis of variance (ANOVA) with the aid of Statistical Package for Social Sciences (SPSS) software, SPSS Inc., Chicago, Standard version 20.0. *P* values < 0.05 were considered significant for differences in mean using the least of significance difference (LSD).

## Figures and Tables

**Table 1:** The activity of AChE versus the percentage (%) inhibition in the brain (cerebrum and cerebellum) and serum after Acacia honey and sodium arsenite administration *in vivo*.

Group	Treatment	AChE activity (*µ*mole/min/g of tissue/mg protein ×10^−2^)	% inhibition of AChE activity	AChE activity (*µ*mole/min/mg protein ×10^2^)	% inhibition of AChE activity
		Brain (cerebrum and cerebellum)		Serum	
1	Distilled water	533.67 ± 7.71^b,c,d^	0.00	1.88 ± 0.32^a,b,c^	0.00

2	20% honey	302.45 ± 5.65^a,c,d^	43.33	1.74 ± 0.43^a^	7.45

3	5 mg/kg sodium arsenite	471.55 ± 11.58^a,b,d^	11.64	1.72 ± 0.19^a^	8.51

4	20% honey + 5 mg/kg sodium arsenite	243.76 ± 4.23^a,b,c^	54.33	1.73 ± 0.54^a^	7.98

Values are presented as mean ± SD; a: statistical significant (*P* < 0.05) as compared with Group 1, b: statistical significance (*P* < 0.05) as compared with Group 2, c: statistical significance (*P* < 0.05) as compared with Group 3, and d: statistical significance (*P* < 0.05) as compared with Group 4.

**Table 2:** The electrolyte levels in brain (cerebrum and cerebellum) after Acacia honey and sodium arsenite administration *in vivo*.

Group	Treatment	Ca^2+^ (mg/dL × 10^−2^)	Na^+^ (mM)	K^+^ (mM)	Cl^−^ (mM)
1	Distilled water	9.20 ± 0.50^b,c,d^	132.00 ± 2.00^b,c,d^	31.08 ± 4.95	134.60 ± 3.78^b,c,d^
2	20% honey	4.00 ± 0.30^a,c,d^	120.00 ± 7.98^a,c,d^	32.77 ± 5.36	122.40 ± 8.11^a^
3	5 mg/kg sodium arsenite	6.66 ± 1.10^a,b,d^	127.60 ± 8.01^a,b,d^	29.38 ± 3.74	124.40 ± 8.11^a^
4	20% honey + 5 mg/kg sodium arsenite	3.00 ± 0.20^a,b,c^	102.00 ± 8.00^a,b,c^	29.39 ± 4.94	123.00 ± 9.58^a^

Values are presented as mean ± SD; a: statistical significance (*P* < 0.05) as compared with Group 1, b: statistical significance (*P* < 0.05) as compared with Group 2, c: statistical significance (*P* < 0.05) as compared with Group 3, and d: statistical significance (*P* < 0.05) as compared with Group 4.

**Table 3:** The electrolyte levels in serum after Acacia honey and sodium arsenite administration *in vivo*.

Group	Treatment	Ca^2+^ (mg/dL)	Na^+^ (mM)	K^+^ (mM)	Cl^−^ (mM)
1	Distilled water	10.86 ± 0.43	144.00 ± 1.52	5.92 ± 1.93	100.33 ± 0.57
2	20% honey	11.95 ± 0.84	143.00 ± 1.41	5.20 ± 1.70	101.25 ± 0.95
3	5 mg/kg sodium arsenite	10.77 ± 0.75	142.66 ± 2.51	6.06 ± 1.40	102.00 ± 0.00
4	20% honey + 5 mg/kg sodium arsenite	11.61 ± 0.60	140.00 ± 0.00	5.45 ± 0.91	101.20 ± 1.09

Values are presented as mean ± SD; a: statistical significance (*P* < 0.05) as compared with Group 1, b: statistical significance (*P* < 0.05) as compared with Group 2, c: statistical significance (*P* < 0.05) as compared with Group 3, d: statistical significance (*P* < 0.05) as compared with Group 4.

**Table 4:** The result of GC-MS analysis of Acacia honey.

S/N	Proposed compounds	Lane (peak)	Similarity index (%)	Proportion (%)
1	2,4-Dihydroxy-5-methylpyrimidine	1	77	3.09
2	Pyrazol-3-one	2	76	6.07
3	5-Hydroxymethylfurfural	3	92	37.96
4	D-Allose	4	94	11.56
5	1,6-Anhydro-beta-D-glucofuranose	5	92	3.88
6	n-Hexadecanoic acid	6	92	2.72
7	Hydrofol acid	7	94	9.44
8	9-Octadecanoic acid-2-hydroxy-1-(hydroxymethyl) ethyl ester	8	81	3.21
9	p-Hydroxybenzoic acids	9	84	1.58
10	Cinnamic acid	10	82	1.08
11	Chrysin	11	82	1.99

## List of Abbreviations

(AChE): Acetylcholinesterase
(ACh): Acetylcholine
(DTNB): 5-5′-Dithiobis 2-nitrobenzoic acid
(AAS): Atomic absorption spectrometry
(GC-MS): Gas chromatography mass spectrophotometry
(CNS): Central nervous system
(ANOVA): Analysis of variance
(LSD): Least of significance difference
(SPSS): Statistical package for social sciences.
